# Hepatic Dysfunction Induced by 7, 12-Dimethylbenz(α)anthracene and Its Obviation with Erucin Using Enzymatic and Histological Changes as Indicators

**DOI:** 10.1371/journal.pone.0112614

**Published:** 2014-11-12

**Authors:** Rohit Arora, Sakshi Bhushan, Rakesh Kumar, Rahul Mannan, Pardeep Kaur, Amrit Pal Singh, Bikram Singh, Adarsh P. Vig, Deepika Sharma, Saroj Arora

**Affiliations:** 1 Department of Botanical and Environmental Sciences, Guru Nanak Dev University, Amritsar, Punjab, India; 2 Sri Guru Ram Das Institute of Medical Sciences and Research, Amritsar, Punjab, India; 3 Department of Pharmaceutical Sciences, Guru Nanak Dev University, Amritsar, Punjab, India; 4 Natural Plant Products Division, CSIR-Institute of Himalayan Bioresource and Technology, Palampur, Himachal Pradesh, India; IDIBAPS - Hospital Clinic de Barcelona, Spain

## Abstract

The toxicity induced by 7, 12-dimethylbenz(α)anthracene (DMBA) has been widely delineated by a number of researchers. This potent chemical damages many internal organs including liver, by inducing the production of reactive oxygen species, DNA-adduct formation and affecting the activities of phase I, II, antioxidant and serum enzymes. Glucosinolate hydrolytic products like isothiocyanates (ITCs) are well known for inhibiting the DNA-adduct formation and modulating phase I, II enzymes. Sulforaphane is ITC, currently under phase trials, is readily metabolized and inter-converted into erucin upon ingestion. We isolated erucin from *Eruca sativa* (Mill.) Thell. evaluated its hepatoprotective role in DMBA induced toxicity in male wistar rats. The rats were subjected to hepatic damage by five day regular intraperitoneal doses of DMBA. At the end of the protocol, the rats were euthanized, their blood was collected and livers were processed. The liver homogenate was analyzed for phase I (NADPH-cytochrome P450 reductase, NADH-cytochrome b5 reductase, cytochrome P450, cytochrome P420 and cytochrome b5), phase II (DT diaphorase, glutathione-S-transferase and γ-glutamyl transpeptidase) and antioxidant enzymes (superoxide dismutase, catalase, guaiacol peroxidise, ascorbate peroxidise, glutathione reductase and lactate dehydrogenase). The level of thiobarbituric acid reactive substances, lipid hydroperoxides, conjugated dienes and reduced glutathione in the liver homogenate was also analyzed. The serum was also analyzed for markers indicating hepatic damage (alkaline phosphatase, serum glutamic oxaloacetic transaminase, serum glutamic pyruvic transaminase, direct bilirubin and total bilirubin). Erucin provided significant protection against DMBA induced damage by modulating the phase I, II and antioxidant enzymes. The histological evaluation of liver tissue was also conducted, which showed the hepatoprotective role of erucin.

## Introduction

**Figure 1:**
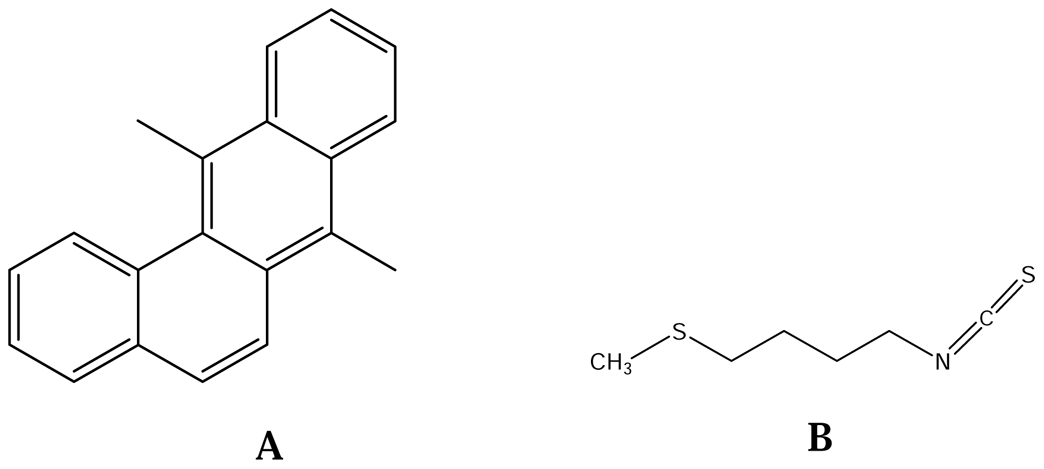
Structural representation of (A) DMBA (7, 12-dimethylbenz(a)anthracene) and (B) erucin.

Polycyclic aromatic hydrocarbons (PAHs) are atmospheric pollutants dispersed abundantly in the ecosystem and are even detected in cooked foods [1], [2]. Amid the various classes of PAHs, 7, 12-dimethylbenz(α)anthracene (DMBA) is a well known carcinogen and immunosuppressor used in rodent models to study cancer [3] (Figure 1A). DMBA is reported to induce mutations by making DNA adducts [4], [5]. Although, it is a well known skin carcinogen, yet many researchers have reported the deleterious effect of DMBA in liver [6], [7], [8]. Liver is the primary site of metabolism and is often prone to damage by xenobiotics. Evidently, liver cancer is the second most common cause of cancer deaths worldwide [9].

Many plant secondary metabolites are known to ameliorate the detrimental effects of xenobiotics and endogenously produced toxins. Glucosinolates (GSLs) are an important class of secondary metabolites, distributed widely in family brassicaceae. The pungent flavor of the consumable plant parts of brassicaceae is due to the presence of GSLs. This pervasive savor is apparently due to activation of transient receptor potential A1 channel in sensory neurons [10]. The hydrolytic products of GSLs such as ITCs are reported for their anticancer, antimutagenic, antimicrobial and herbicidal properties [11], [12]. Among the various ITCs, sulforaphane has been widely explored for its anticancer properties and has been included in the clinical trials on human subjects [13], [14]. Sulforaphane is structurally related to erucin (1-isothiocyanato-4-methylsulfinyl-butane), an ITC available largely in the seeds of *Eruca sativa* (Mill.) Thell. (Figure 1B). It is a hydrolytic product of glucoerucin and is known to induce cell cycle arrest and down regulate androgen receptor signaling [15]. Erucin has been reported to act against pulmonary carcinogen by promoting *in situ* detoxification of their toxic products in rats [16]. The present study was designed to investigate the role of erucin in modulating the DMBA induced alterations in phase I, II, antioxidant enzymes and serum parameters. To best of our knowledge this is the first report of erucin to counteract the damaging effects of DMBA in rats.

## Results

### Isolation of Erucin

Erucin was successfully isolated from the extract of *Eruca sativa* (Mill.) Thell. The purified compound showed a purity of ≥99% in GC-FID and it was further analyzed using mass spectrometry, ^1^H and ^13^C NMR spectra at 600 MHz. 1H NMR (600 MHz, CDCl_3_): δ 1.64–1.78 (m, 4H), 2.04 (s, 3H), 2.48 (t, j = 2.9 Hz, 2H), 3.5 (t, j = 6.3 Hz, 2H). ^13^C NMR (400 MHz, CDCl_3_): δ 15.41, 25.83, 28.85, 33.27, 44.76, 130.09 (Figure S1). The NMR data was compared with the available literature for further confirmation [17].

### Effect of Erucin on DMBA Induced Alterations in Hepatic Phase I Enzymes

**Figure 2:**
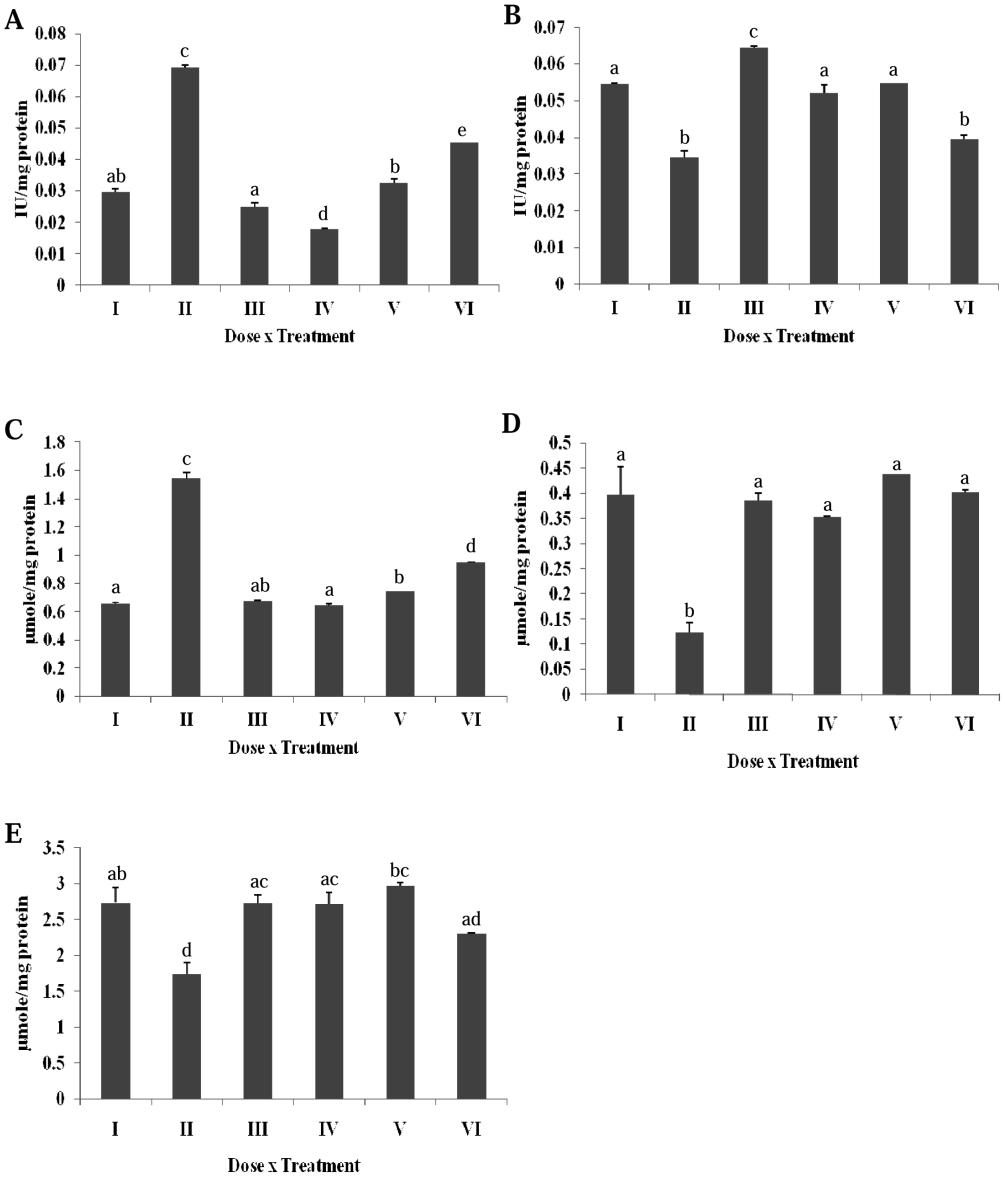
Effect of erucin on DMBA induced alterations in hepatic phase I enzymes (A) NADH-cytochrome b5 reductase, (B) NADPH-cytochrome P450 reductase, (C) Cytochrome b5, (D) Cytochrome P450, (E) Cytochrome P420. Treatment groups with same alphabet show no significant difference, while the groups with different alphabets show significant difference at p≤0.05. Error bars indicate SEM (n = 6).

A significant increase in the NADH cytochrome b5 reductase activity of DMBA treated rats was observed as compared to the control (Figure 2A). The specific activity in the other groups treated with erucin (gp IV, V and VI) was reduced towards the normal level. In case of NADPH cytochrome P450 reductase, a significant decrease was noted in the liver homogenate of DMBA treated group (Figure 2B). All the treatment groups significantly accorded protection from DMBA (gp IV, V and VI), but group VI witnessed a decreased enzyme activity nearing DMBA treatment.

The conversion of DMBA into carcinogenic form was clearly represented by the two fold significant increase of cytochrome b5 enzyme in the DMBA treated group in contrast to the untreated control (Figure 2C). All the other groups (gp IV, V and VI) rendered significant protection against the toxic effects of DMBA. A significant reduction in cytochrome P450 enzyme was observed in DMBA treated group as compared to untreated control. This reduction was later normalized at all dose levels of erucin (gp IV, V and VI) (Figure 2D). The cytochrome P420 enzyme was significantly reduced in the DMBA treated rats as compared to the untreated group (Figure 2E). Treatment with erucin at all dose levels (gp IV, V and VI) witnessed significant protection against DMBA treatment.

### Effect of Erucin on DMBA Induced Alterations in Hepatic Phase II Enzymes

**Figure 3:**
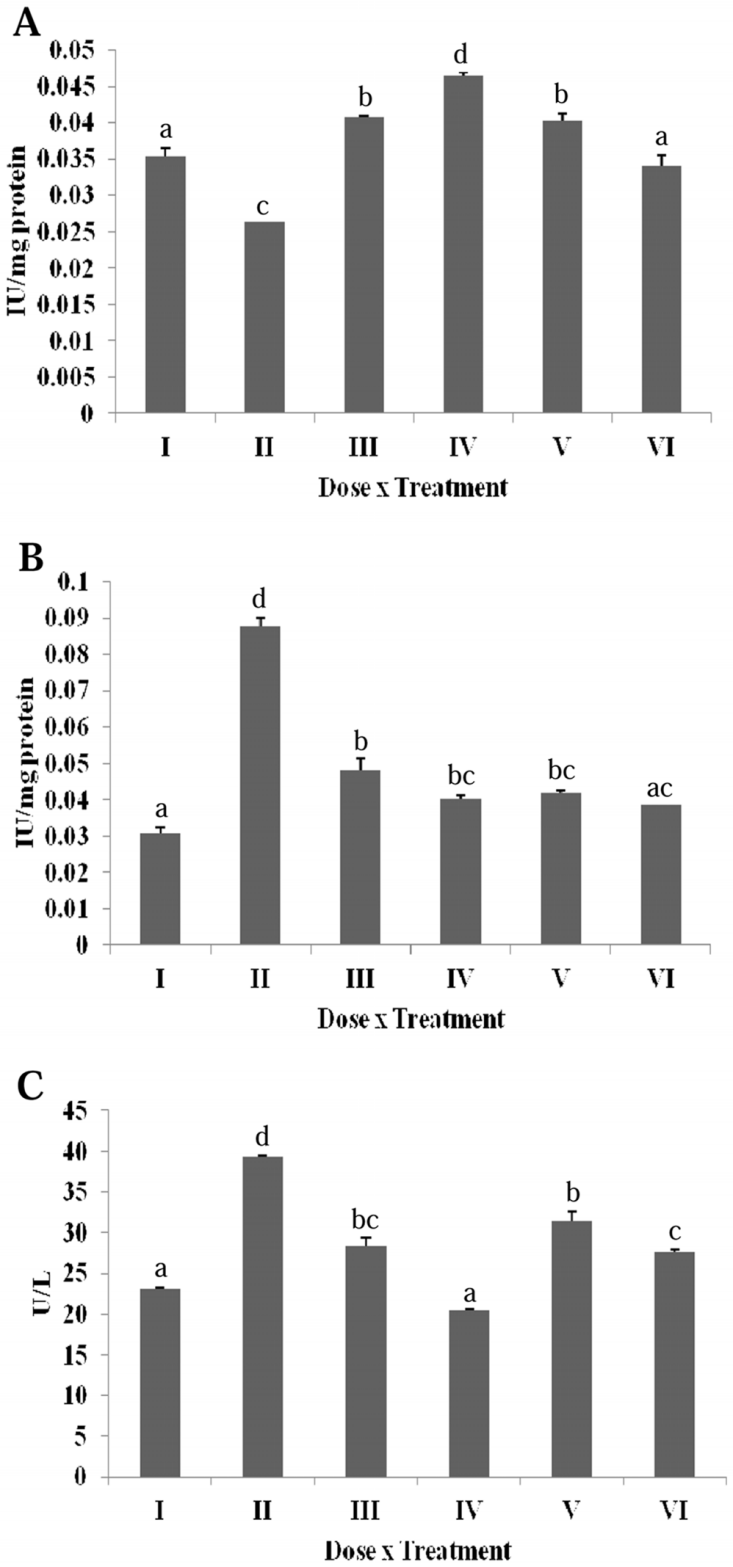
Effect of erucin on DMBA induced alterations in hepatic phase II enzymes (A) Glutathione-S-transferase, (B) DT-diaphorase, (C) γ-glutamyl transpeptidase. Treatment groups with same alphabet show no significant difference, while the groups with different alphabets show significant difference at p≤0.05. Error bars indicate SEM (n = 6).

The GST activity of liver homogenate was significantly reduced in the DMBA treated rats in contrast to the untreated group (Figure 3A). Erucin treatments at all dose levels in gp IV, V and VI were able to counteract the damaging effects of DMBA and successfully increased the specific activity of GST.

The specific activity of DTD in liver homogenate was found to be three fold higher in the DMBA treated groups in comparison to the normal untreated group (Figure 3B). A decrease of DTD activity in all the treatment groups (gp IV, V and VI) was observed. The toxicity of DMBA was clearly observed, as the GGT activity was found to be significantly higher in the DMBA treated rats compared to untreated control (Figure 3C). Erucin was successful in counteracting the damaging effect of DMBA in all the treatment groups (gp IV, V and VI) and brought back the GGT levels to normalcy.

### Effect of Erucin on DMBA Induced Alterations in Hepatic Antioxidant Enzymes

**Figure 4:**
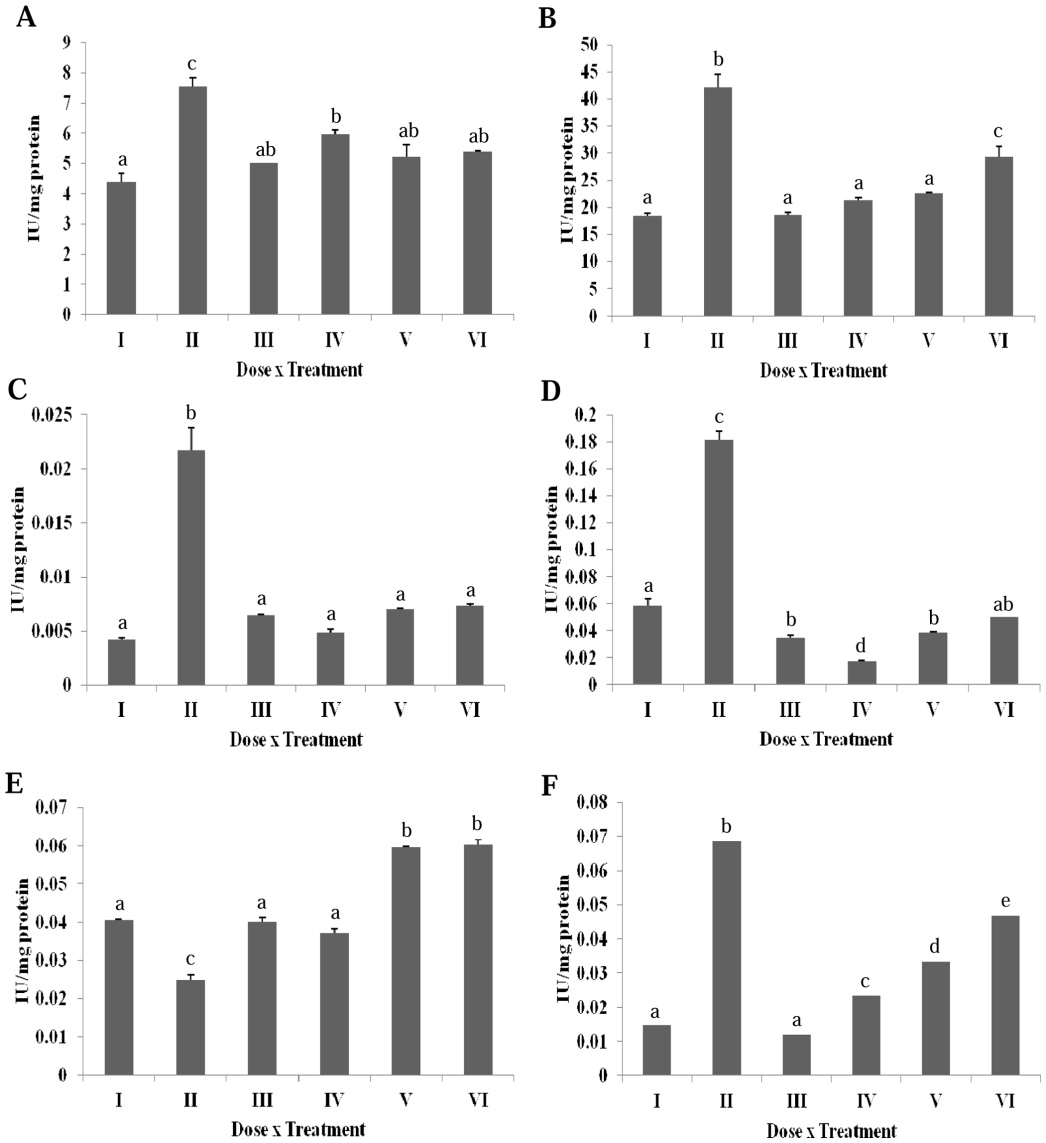
Effect of erucin on DMBA induced alterations in hepatic anti-oxidant enzymes (A) Superoxide dismutase, (B) Catalase, (C) Guaiacol peroxidase, (D) Ascorbate Peroxidase, (E) Glutathione reductase, (F) Lactate dehydrogenase. Treatment groups with same alphabet show no significant difference, while the groups with different alphabets show significant difference at p≤0.05. Error bars indicate SEM (n = 6).

A significant increase in SOD activity was observed in the DMBA treated group as compared to untreated group (Figure 4A). Rest all of the treatment groups were found to have SOD activity equal to the untreated groups. A significant decrease in the CAT activity of the untreated rats was observed as compared to the DMBA treated rats (Figure 4B). Erucin treatment at all dose levels (gp IV, V and VI) brought the CAT levels near control group I. The GPOX activity was found to be significantly higher in the DMBA treated group in contrast to the untreated group (Figure 4C). All the three erucin treatment groups *viz*. IV, V and VI significantly mitigate the toxic effects of hydrogen peroxide radical produced by DMBA. The specific activity of APOX enzyme was significantly enhanced in the DMBA treated group as compared to the untreated group (Figure 4D). The activity of APOX was significantly reduced in erucin treatment groups IV, V and VI as compared to the DMBA treated groups.

The reduction of oxidized glutathione to reduced glutathione by glutathione reductase (GR) is another mechanism of body for scavenging free radical. The GR activity was observed to be significantly reduced in DMBA treated groups compared to the untreated group (Figure 4E). The treatment with erucin (gp IV, V and VI) led to a dose dependent significant increase of the GR activity as compared to the DMBA treated group. Membrane integrity is measured by the lactate dehydrogenase (LDH) activity of the liver homogenate. A fivefold significant increase in the LDH activity was observed in the DMBA treated groups in contrast to untreated rats (Figure 4F). The treatment with erucin in gp IV, V and VI was successful in counteracting the damaging effect of DMBA. An increase in the LDH activity was observed at the highest concentration of erucin, pointing towards a slight toxicity of erucin at higher concentration.

### Effect of Erucin on DMBA Induced Alteration on Other Oxidative Stress Parameters in Liver Homogenate

The lipid molecules under the influence of reactive species are broken down into conjugated dienes that are readily converted into lipid hydroperoxides and thiobarbituric acid reactive substances (TBARS). The TBARS content of DMBA was significantly higher than the untreated control (Table 1). The TBARS value of the treatment groups IV, V and VI was significantly reduced in comparison with the DMBA treated rats. The value of conjugated dienes was significantly higher in DMBA treated group in contrast to the untreated group (Table 1). All the erucin treatment groups *viz*. IV, V and VI were successful in restraining the harmful effects of DMBA. The lipid hydroperoxide content of DMBA treated group was lower than the untreated group (Table 1). The treatment groups IV, V and VI witnessed a half decrease in contrast to the untreated group.

**Table 1:** Effect of DMBA and erucin alone and in combination on lipid and reduced glutathione content.

Groups	TBARS (µmoles MDA equivalent/g of tissue) ± SE	Conjugated dienes (nmoles/mg protein) ± SE	Lipid Hydroperoxide (mM H_2_O_2_ equivalents/g of tissue) ± SE	Reduced Glutathione content (µmoles of SH content/g of tissue) ± SE
**I**	20.099±0.065^a^	549.979±01.293^abc^	7.103±0.068^c^	438.380±34.163^a^
**II**	44.384±0.139^d^	798.666±05.783^d^	3.433±0.230^d^	281.219±01.004^b^
**III**	13.518±0.672^c^	599.251±09.055^a^	5.046±0.156^a^	497.268±12.844^a^
**IV**	15.687±1.594^bc^	455.225±21.158^e^	4.189±0.094^b^	301.576±09.645^b^
**V**	17.655±0.615^ab^	534.621±04.719^b^	5.668±0.191^a^	497.183±02.335^a^
**VI**	35.635±0.571^e^	621.326±04.041^c^	4.269±0.152^b^	433.966±09.953^c^

The reduced glutathione is an important enzyme, responsible for the reduction of oxyradicals through conjugation and reduction reactions [6]. The reduced glutathione was significantly reduced in DMBA treated group as compared to untreated group (Table 1). All erucin treatment groups *viz*. IV, V and VI significantly elevated the reduced glutathione content as compared to the DMBA treated group.

### Effect of Erucin on DMBA Induced Alterations in Serum Enzymes

**Figure 5:**
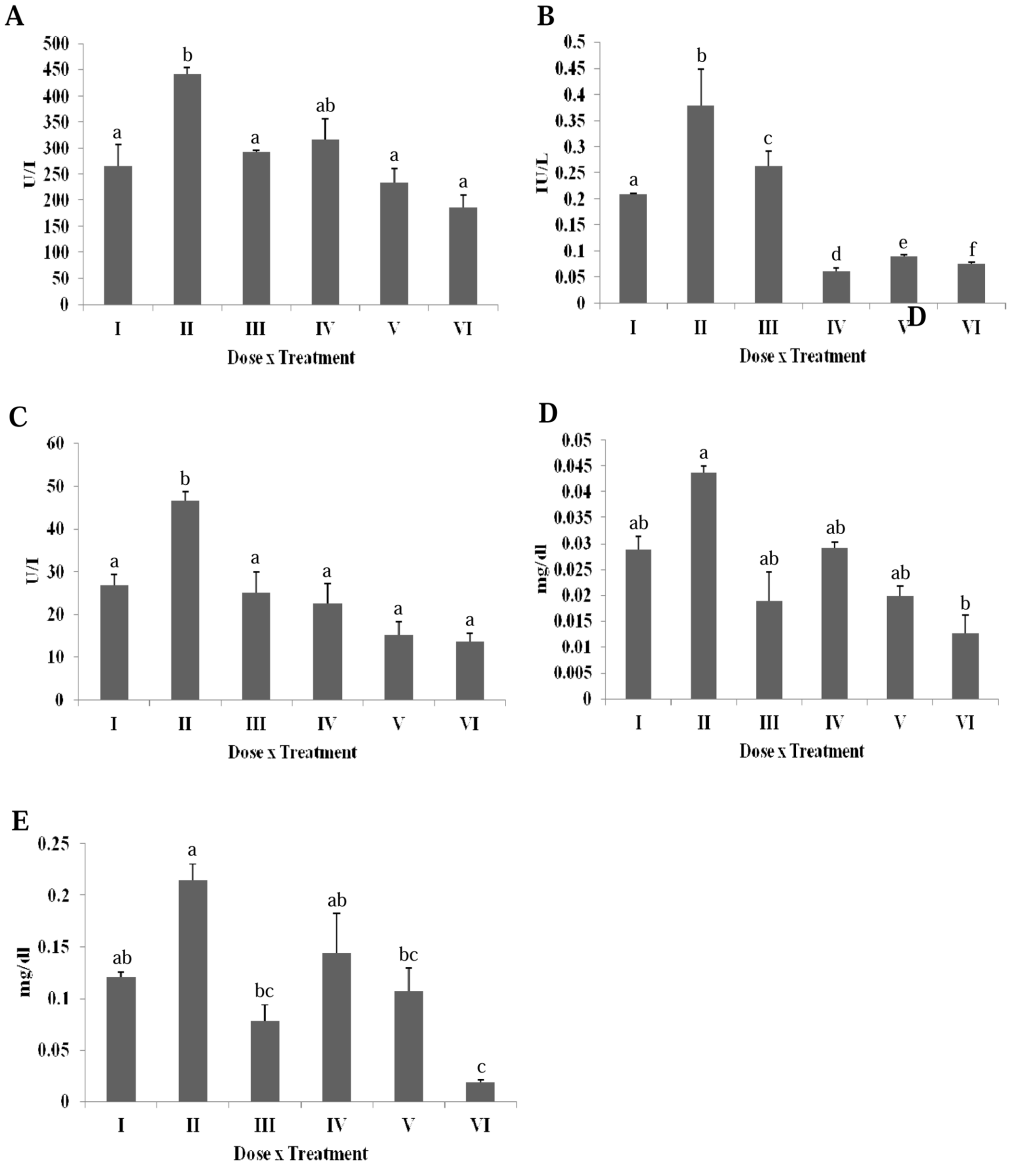
Effect of erucin on DMBA induced alterations in serum enzymes (A) Alkaline phosphatase, (B) Serum glutamic oxaloacetic transaminase, (C) Serum glutamic pyruvic transaminase, (D) Direct bilirubin, (E) Total bilirubin. Treatment groups with same alphabet show no significant difference, while the groups with different alphabets show significant difference at p≤0.05. Error bars indicate SEM (n = 6).

DMBA significantly increased the serum alkaline phosphatase (ALP) level as compared to the untreated group (Figure 5A). A significant decrease in the ALP activity was observed following the erucin treatment (gp IV, V and VI). A significant increase in the serum glutamic oxaloacetic transaminase (SGOT) or aspartate transaminase (AST) in DMBA treated group was observed in comparison to the untreated group (Figure 5B). A significant decrease in the SGOT level was seen following the erucin treatment (gp IV, V and VI). Serum glutamic pyruvic transaminase (SGPT) or alanine transaminase (ALT) analysis revealed a significant increase in the case of DMBA treatment and a reduction following the erucin treatment (gp IV, V and VI) (Figure 5C). Bilirubin is the breakdown product of haeme, found in haemoglobin, the prime constituent of red blood cells (RBCs). DMBA showed an increase in direct and total bilirubin level compared to the normal range, indicating a decreased bilirubin clearance from blood (Figure 5D and 5E). On the other hand, the higher concentrations of erucin significantly decreased both the direct and total serum bilirubin level in contrast to the DMBA treated group.

### Histological Analysis

**Figure 6:**
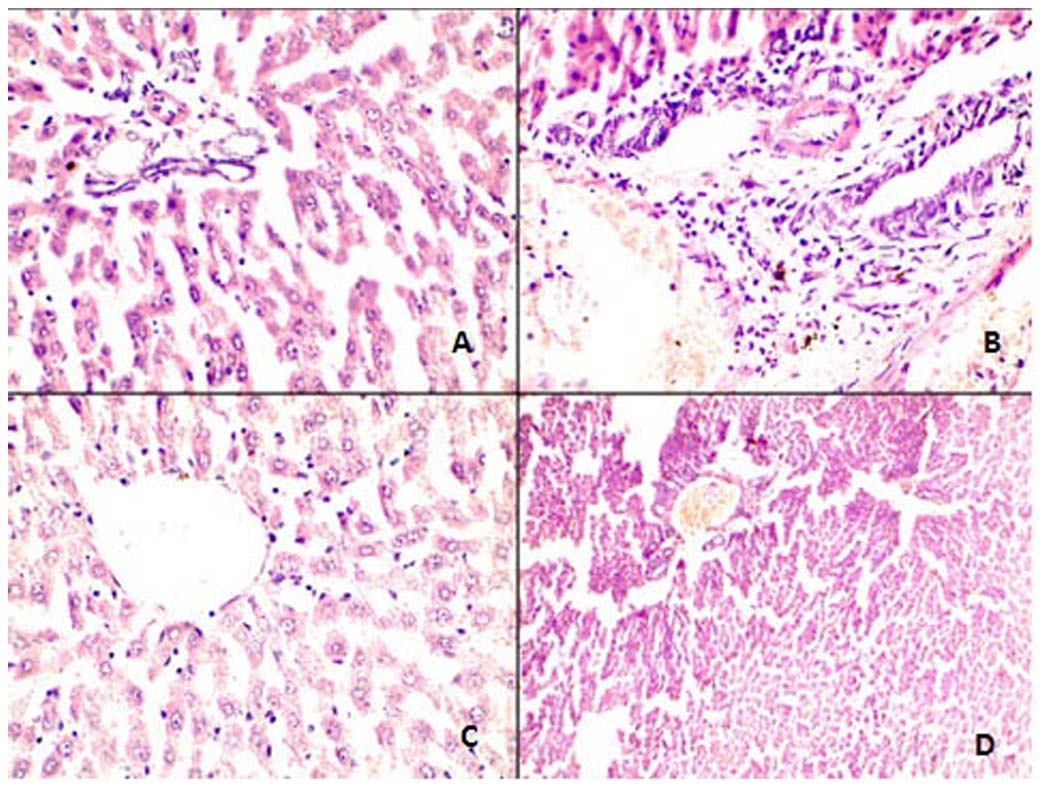
Comparative changes noted on histology- Normal Vs Abnormal at H&E 400 X. (**A**) Normal portal triad comprising of hepatic duct, artery and portal vein without any inflammatory infiltrate.; (**B**) Portal tracts showing mild to moderate periportal inflammation by mononuclear inflammatory cells; (**C**) Normal central vein which is surrounded by unremarkable looking hepatocytes maintaining their normal trabecular architecture and orientation; (**D**) Central vein is dilated and congested and shows loss of normal trabecular architecture along with focal necrosis.

As evident from the necroinflammatory scores, the DMBA treated group incurred the highest damage (score 5/18), as seen through the various structural changes (Figure 6, Table 2). Untreated control and the erucin alone treatment group III had a zero total score, confirming no negative effect of erucin at this dose. The highest dose of erucin alone showed the symptoms of focal lytic necrosis and partial portal inflammation, adding to the total score of 2/18. The other treatment groups in which erucin was given in combination with DMBA, a slight tissue change was seen in both group V (score 1/18) and VI (score 2/18).

**Table 2:** Modified hepatic activity index grading: Necroinflammatory scores.

Groups	Periportal or interface hepatitis (Piecemeal Necrosis)	Confluent Necrosis	Focal (spotty) Lytic Necrosis, Apoptotic and Focal Inflammation	Portal Inflammation	Total Score
Control					
**I**	0	0	0	0	**0/18**
**II**	1	0	2	2	**5/18**
**III**	0	0	0	0	**0/18**
**IV**	0	0	0	0	**0/18**
**V**	0	0	1	0	**1/18**
**VI**	0	0	1	1	**2/18**

## Discussion

Modernization and an ever increasing demand to meet its pace is the key root cause of the escalating pollution. The increasing pollution leads to the worldwide production of genotoxic PAHs. DMBA is one of the most common and highly toxic PAH, which is a strong immunosuppressor and forms covalent DNA adducts [18]. The carcinogenic and mutagenic effect of DMBA requires its metabolic activation by mixed function oxidases. The hydroxylation of DMBA at 7-methyl group is a crucial step towards its carcinogenesis [19]. Further metabolism of DMBA leads to formation of a wide range of metabolites with varying toxicity. Among these, trans-3,4-dihydrodiol-1,2-epoxide is the carcinogenic product of DMBA [20], [21]. These metabolic products of DMBA, when present inside body, hampers ROS-antioxidant balance by overproduction of free radicals and the body in turn reacts by modulating activities of antioxidant enzymes to curb the damaging effects of an increased ROS [22]. In the present study, ITCs have been used to investigate their protective activity against DMBA induced damage in male wistar rats. ITCs are known for their ability to inhibit the DNA-adduct formation and upregulating the cellular antioxidant enzymes to inhibit the cellular proliferation [23], [24]. Most of the ITCs are monofunctional inducers, since they downregulate the phase I enzymes and upregulate the phase II enzymes [23]. One of the most important ITCs is sulforaphane which is currently under phase trials for its anticancer properties. Sulforaphane which when ingested is inter-converted into erucin [4]. We isolated erucin or 4-methylthiobutyl isothiocyanate from the seeds of *Eruca sativa* (Mill.) Thell. with a purity of ≥99%. The purity of erucin was estimated by GC-FID, mass spectra, NMR (^1^H and ^13^C) and available literature. Male wistar rats were chosen for the present study since a number of manuscripts are available showing the damaging effect of DMBA on the liver of these rats [25], [26].

DMBA is readily metabolized in the living system through some specialized modulatory enzymes. These enzymes are termed as the phases of detoxification. Phase I enzymes introduce reactive and functional groups, thus increasing the polarity of drug. The three most important mechanisms responsible for enhancing the polarity are oxidation, reduction and hydrolysis [9]. A significant increase in the NADPH cytochrome P450 reductase, cytochrome P450 and cytochrome P420 was seen in all treatment groups compared to the DMBA treated group. A lower level of cytochrome P450 in comparison to cytochrome P420, might be due to the conversion of the active CYP enzyme into the inactive form (cytochrome P420) [27]. Cytochrome b5 and NADH cytochrome b5 reductase was significantly increased in the DMBA treated group in contrast to the untreated group, suggesting its potential role in converting DMBA into its electrophillic form. Cytochrome b5 is an important enzyme and it is proposed that this cytochrome donates an electron to monoxygenase cycle. On the other hand, P450 monoxygenases are responsible for releasing reactive species such as superoxide radicals and thus converts the drug into its electrophilic form [28].

Phase II enzymes convert the metabolically active forms of drug into less active form, which are readily excreted from the body [9]. GST is an important enzyme, which neutralizes the active sites of electrophiles and initiates detoxification process [8]. A decreased GST level in DMBA group as compared to the control may be due to an enhanced radical accumulation by this group. All the other groups; erucin alone and in combination with DMBA resulted in a gradual increase of GST and thus normalized the DMBA induced toxicity. DTD is another important phase II enzyme responsible in the 2-electron reduction of quinones and thus participates in electron transport chain [29]. An increase in the DTD, as in the case of DMBA treated group, is generally seen in certain tumors such as liver cancer [29]. The treatment with erucin reduced the DTD value as a result of the conversion of quinines into hydroquinone and their further elimination by antioxidative system. GGT is an enzyme responsible for transfer of γ-glutamyl moiety to an acceptor. Its enhanced level is usually a symptom of oxidative stress and is also associated with the risk of cancer development [30]. An increased GGT activity in the DMBA treated group is countered by the protective role of erucin as seen by reduction in all the treatment groups.

Apart from the phase I and II enzymes, the ROS generated by DMBA is also counteracted by the *in vivo* antioxidative enzymes. The most toxic and reactive species is the superoxide radical and is the key component responsible for the generation of other free radicals and thus propagating chain reaction. The increased SOD content might be due to enhanced oxidative stress induced by DMBA. Administration of erucin reduced the SOD content, pointing towards the antioxidant properties of erucin. SOD converts the toxic superoxide radicals into less toxic hydrogen peroxide radicals. An enhanced level of catalase, GPOX and APOX was observed in the DMBA treated group. The treatment with erucin alone as well as in combination with DMBA caused a significant decrease in these enzymes as compared to the DMBA treated rats. An enhanced antioxidative defence system of treatment groups was observed owing to the synergistic antioxidative property of erucin. Following the final normalization step of the Halliwell-Asada pathway, the GR enzyme activity of liver homogenates was assessed. The GR activity in DMBA treated rats was found to be significantly lower in comparison to the untreated group. This decrease might be due to its inability to reduce its oxidized glutathione. The GR enzyme activity in all the other treatment groups was found to be higher than the DMBA group, suggesting their role in maintaining the basal level of GR. The results obtained were found to coincide with those of GST. LDH is a very important enzyme; an increase in its activity suggests the cellular damage. A six to seven fold increase in the LDH enzyme activity was observed in DMBA treated rats as compared to untreated group. It might points towards the hepatic injury caused by DMBA; the level of this enzyme showed a significant decrease after the treatment with erucin alone as well as in combination with DMBA.

Lipids are important biomolecules and one of the key constituent of cellular membranes. The lipid molecules are broken down into a number of products such as conjugated dienes, TBARS and lipid hydroperoxides by ROS. An elevated level of TBARS and conjugated dienes was observed in the DMBA treated group, suggesting towards the increased ROS generation. A significant reduction in the TBARS and conjugated dienes was found in erucin treated rats. The increased lipid hydroperoxide content in untreated group indicated a possible conversion of lipids either into conjugated dienes or TBARS. Reduced glutathione is an important antioxidant enzyme which reduces the oxyradicals through reduction and conjugation reaction [8]. The level of reduced glutathione was lowered in the DMBA treated group, indicating the increased consumption of this enzyme for the counteraction of the reactive oxygen species. Activity of reduced glutathione was enhanced following the treatment by erucin, indicating the possible antioxidant role of erucin.

In addition to the direct stress markers, the indirect markers of injury i.e. the serum markers of hepatic injury were also studied. Serum is a part of blood and circulates throughout the body. Injuries occurring due to DMBA, will not only be revealed by the liver homogenate, but will also be reverberated by the various serum markers. Alkaline phosphatase is a very important serum marker responsible for removing phosphate groups from various molecules [31]. An enhanced activity of alkaline phosphatase in the DMBA treated group suggests towards the liver damage. All the other treatment groups witnessed a significant decrease in the alkaline phosphatase levels. SGOT, commonly found in liver, catalyzes the reversible transfer of α-amino group between glutamate and aspartate [32]. An increased SGOT level in DMBA treated group was observed, pointing towards hepatic injury. A dose dependent decrease in the SGOT levels was observed following erucin treatment. SGPT is another important enzyme responsible for the reversible transfer of amino group from L-alanine to α-ketoglutarate [33]. An elevated level of SGPT in DMBA treated group suggested liver damage. The erucin treatment groups witnessed a significant protection from DMBA induced toxicity. Bilirubin is the breakdown product of haeme, found in haemoglobin, the prime constituent of red blood cells (RBCs). Both direct (conjugated) as well as indirect (unconjugated) bilirubin was studied, the difference being the conjugation of bilirubin with glucuronic acid in liver and thus making it water soluble [34]. The level of both direct and indirect bilirubin was significantly increased in the DMBA treated group and its enhanced level indicated haemolysis or liver injury. The level of bilirubin was normalized by all three erucin treatment groups.

The histological alterations in all the treatment groups *viz*. untreated, DMBA and erucin alone and in combination were studied. Normal physiology (Figure 6A) with no change in liver histology was observed in untreated group and in group III (erucin given alone). The same physiology was observed in group IV rats treated with 15 mg/kg bw erucin. On the other hand, the maximum damage was observed in DMBA treated rats, showing mild periportal necrosis along with mild portal inflammation and two to four foci per 10× objective. DMBA is toxic and the production of ROS resulted in the chain reaction, leading to damage incurred on lipids along with other biomolecules, resulting in the formation of mild periportal necrosis (Figure 6B), two to four foci per 10× objective and moderate portal inflammation. The treatment with 35 mg/kg body weight of erucin in addition with DMBA in group V showed only one focus or less per 10× objective and had slight damage in comparison with the untreated group. Group VI showed one focus per 10× objective (Figure 6C) and mild periportal inflammation (Figure 6D). This group revealed a slight toxicity of erucin at this concentration, but the damage incurred was lower as compared to group V. No confluent necrosis was observed in any group. All the histopathological changes were found to correlation with the results obtained from liver enzymes and serum parameters.

## Conclusion

**Figure 7:**
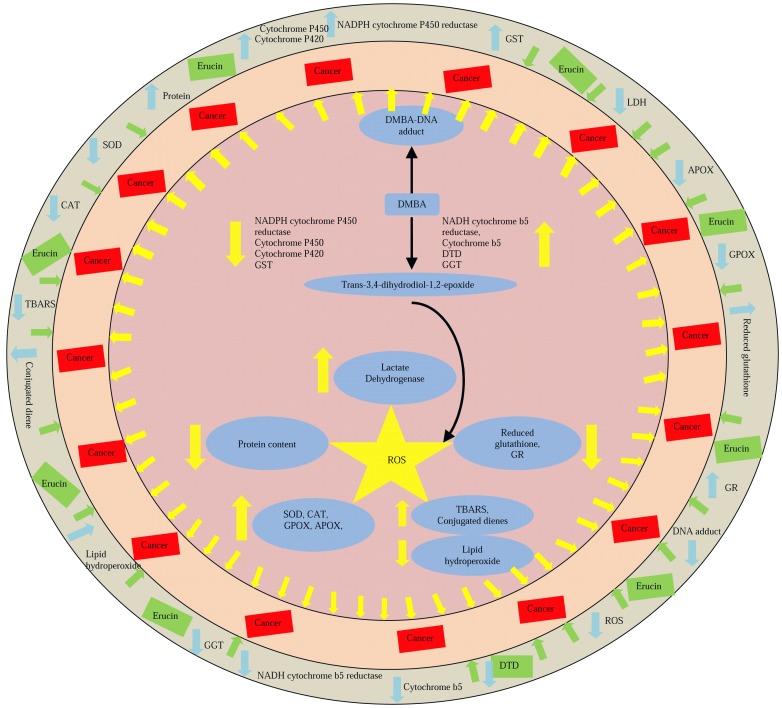
Depicts the graphical representation of modulation of different parameters in response to the interaction of DMBA (7, 12-dimethylbenz(a)anthracene) and erucin in the *in vivo* system. ↑ -Shows increase in the activity: ↓ -Shows decrease in the activity. Yellow arrow represents general effect of reactive oxygen species. Blue arrows represents effect of DMBA and green arrows represents the effect of erucin.

It is concluded that lower doses of erucin provided protective against 7,12-dimethylbenz(a)anthracene induced damage by modulating phase I, II and antioxidant enzymes (Figure 7). Moreover, the serum parameters were also normalized by erucin administration. All these biochemical changes were supported by histological analysis which showed normal hepatic architecture in erucin fed rats.

## Materials and Methods

### Animal Ethics Statement

Permission for working on rat model was granted by Committee for the Purpose of Control and Supervision of Experiments on Animals (CPCSE), Ministry of Environment and Forests, Government of India (226/CPCSEA).

### Chemicals

Dichloromethane, ethyl acetate, hexane, sodium bicinchoninate, 7, 12-dimethylbenz(α)anthracene, 5-5-dithiobisnitrobenzoic acid (DTNB), sodium dithionate, L-γ-glutamyl-4-nitroanilide, H_2_O_2_, and malondialdehyde (MDA) were purchased from Sigma-aldrich, Bangalore, India. Xylenol orange, ammonium ferrous sulfate, cyclohexane, 2, 6-dichlorophenol-indophenol (DCPIP), reduced nicotinamide adenine dinucleotide (NADH), reduced nicotinamide adenine dinucleotide phosphate (NADPH), bovine serum albumin (BSA), potassium ferricyanide, 1-chloro-2,4-dinitrobenzene (CDNB), glycylglycine, reduced glutathione (GSH), oxidized glutathione (GSSG), ascorbic acid, guaiacol, Triton-X, nitroblue tetrazolium chloride (NBT), hydro-xylamine hydrochloride, and pyruvate were purchased from Himedia Laboratories, Mumbai, India. DMBA (7, 12- dimethylbenz(α)anthracene) was obtained from Sigma-aldrich, Bangalore, India. Autopack kit for analyzing bilirubin was purchased from Beacon Diagnostics, Gujarat, India, while alkaline phosphatase, serum glutamic oxaloacetic transaminase and serum glutamic pyruvic transaminase autopack kits were purchased from Delta Labs, Sindhudurg, India. Rest of the chemicals used in the present study were of analytical grade.

### Isolation of erucin

Seeds of *Eruca sativa* (Mill.) Thell. were purchased from Sri Karan Narendra College of Agriculture, Jobner, Rajasthan (India). The seeds were washed and dried to remove any dust particles. These were later air dried and stored at −20°C until extraction. The extraction of ITCs from these seeds was performed using a slightly modified hydrodistillation protocol [12], [35]. The crushed seeds (100 g) were immersed in round bottom flask containing 1000 ml distilled water. It was subjected to boil using heating mantle and the vapors along with the volatile ITCs were condensed and collected in the inner tube. It was later concentrated at 30°C under vacuum using rotary evaporator (Buchi R210). The obtained seed extract was then further fractionated using column chromatography, to obtain purified erucin (confirmed using GC, ^1^H and ^13^C NMR).

### Animals and Treatment

**Figure 8:**
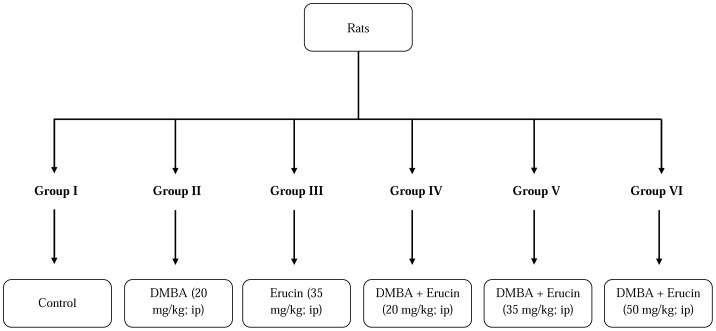
Treatment schedule of DMBA (7, 12-dimethylbenz(a)anthracene), erucin alone and in combination of both DMBA and erucin.

Male albino wistar rats (8–12 weeks old) weighing 150–240 g were used in the present study. The rats were housed in polypropylene cages using paddy husk bedding at 25±2°C temperature, with a 12 h light: 12 h dark cycle on a daily basis and were fed a standard pellet diet and had water ad libitum. They were acclimatized for 2 weeks before the onset of the experiment and were housed in the animal house of Guru Nanak Dev University (GNDU), Amritsar, as per the guidelines of Committee for the Purpose of Control and Supervision of Experiments on Animals (CPCSE), Ministry of Environment and Forests, Government of India (226/CPCSEA). Rats were randomly divided into six groups having six in each group (Figure 8). The different concentrations of erucin (20 mg/kg, 35 mg/kg and 50 mg/kg body weight) and DMBA (20 mg/kg body weight) were prepared in corn oil, given to rats intraperitoneally. The treatments were given for five days at regular intervals of 24 h and the experiment was terminated on sixth day. The five day treatment of DMBA was selected following the reported methods [36], [37]. The animals were euthanized at the end of the protocol.

### Preparation of liver homogenates

Preparation of homogenates was carried out on ice. Before excising the liver it was perfused immediately with ice chilled 0.9% NaCl and thereafter carefully removed, made free of unrequired tissue. It was then rinsed in chilled 0.15 M Tris–KCl buffer (0.15 M KCl+10 mM Tris–HCL, pH 7.4). The liver was then dried, weighed quickly and homogenized in ice cold 0.15 M Tris-KCl buffer to yield 10% (w/v) homogenate. 0.5 ml of this homogenate was precipitated with 5% trichloroacetic acid, centrifuged at 2000 rpm and the supernatant was used for the estimation of reduced glutathione content. The homogenate, after discarding any lipid layer, was used for the estimation of phase I and II enzymes like NADPH-cytochrome P450 reducatse, NADH-cytochrome b5 reductase, Cytochrome P450, Cytochrome P420, Cytochrome b5 & DT diaphorase (DTD), γ-Glutamyl Transpeptidase (GGT) and glutathione-S-transferase (GST), lipid peroxidation in terms of TBARS, conjugated dienes and lipid hydroperoxide content and antioxidative enzymes viz., superoxide dismutase (SOD), catalase (CAT), guiacol peroxidase (GPOX), ascorbate peroxidase (APOX), glutathione reductase (GR), lactate dehydrogenase (LDH) respectively.

### Biochemical Studies

#### Effect of Erucin on DMBA Induced Alterations in Hepatic Phase I Enzymes

NADPH-cytochrome P450 reductase (E.C.1.6.2.4) activity was performed following the method by Omura and Takasue [38] and activity of NADH-cytochrome b5 reductase (E.C.1.6.2.2) was assayed using method described by Mihara and Sato [39] with slight modifications. For NADPH-cytochrome P450 reductase, 0.1 mM NADPH was added to 0.3 M phosphate buffer (pH = 7.5) followed by 0.2 mM potassium ferricyanide and tissue homogenate in a final volume of 1 ml and absorbance was taken at 340 nm. The enzyme activity was calculated using extinction coefficient 6.22 mM^−1^cm^−1^. The activity of NADH-cytochrome b5 reductase was calculated using reaction mixture comprised of 0.1 M potassium phosphate buffer (pH = 7.5), 0.1 mM NADH, 1 mM potassium ferricyanide and homogenate in a final volume of 1 ml. Absorbance was read at 420 nm. The enzyme activity was calculated using extinction coefficient of 1.02 mM^−1^cm^−1^.

Cytochrome P420 content of the tissue was determined using the carbon monoxide difference spectra as obtained by following the methodology of Choi et al. [40]. Cytochrome P420 concentration was determined from the change in absorbance at 420 nm and 490 nm using an absorption coefficient of 111 mM^−1^cm^−1^. Cytochrome P450 content of the tissue was determined using the carbon monoxide difference spectra as obtained by following the methodology of Choi et al. [40]. Cytochrome P450 concentration was determined from the change in absorbance at 450 nm and 490 nm using an absorption coefficient of 91 mM^−1^cm^−1^. The estimation of cytochrome b5 content was done by recording spectral difference between 424 nm and 409 nm of oxidized and NADH reduced homogenate using an absorption coefficient of 185 mM^−1^cm^−1^ following the method given by Omura and Sato [41].

#### Effect of Erucin on DMBA Induced Alterations in Hepatic Phase II Enzymes

Glutathione-S-transferase (E.C. 2.5.1.18) activity was determined spectrophotometrically as described by Habig et al. [42]. The reaction was started by adding 0.1 ml of tissue homogenate to reaction mixture (0.1 M phosphate buffer (pH = 6.5), 30 mM CDNB and 30 mM reduced glutathione), followed by incubation at 37°C for 3 minutes. The absorbance was read at 340 nm for 3 minutes. The glutathione-S-transferase activity was calculated using an extinction coefficient of 9.6 mM^−1^cm^−1^.

NAD(P)H:quinone oxidoreducatse (EC 1.6.99.2) also known as DT-diaphorase was measured according to the method described by Ernster [43] with slight modifications. The reaction mixture comprising of 0.05 M Tris Buffer (pH = 7.5), NADH (0.3 mM), DCPIP (0.4 mM) and BSA (0.7%) was mixed with homogenate and the absorbance was measured at 600 nm. The activity was calculated using an extinction coefficient 21 mM^−1^cm^−1^.

γ- glutamyl transpeptidase (EC 2.3.2.2) enzyme plays an important role in detecting liver related disorders including tumors. The enzyme activity was determined following method described by Szasz [44]. To a 1 ml of working reagent (prepared by mixing 5 mM L-γ-glutamyl-4-nitroanilide in 0.1 N HCl (R1) and 0.1 M of glycylglycine in 0.1 M Tris Buffer (pH = 8.6) (R2) in the ratio of 1∶4), 0.1 ml of homogenate was added followed by incubation for 1 minute. Absorbance was measured at 405 nm. The activity of γ-glutamyl transpeptidase was calculated by multiplying mean change in absorbance per minute with a factor (1158).

#### Effect of Erucin on DMBA Induced Alterations in hepatic Antioxidant Enzymes in Liver

Superoxide dismutases (EC 1.15.1.1) are enzymes responsible for scavenging or dismutation of superoxides following the method given by Kono [45] with slight modifications. The percent inhibition of the sample (y) was calculated using the formula: [(ΔAbs/min_(Blank)_- ΔAbs/min_(Sample)_)/ΔAbs/min_(Blank)_]. A 50% inhibition was calculated as per the formula: [(50×70)/y] and the specific activity was calculated using protein content. Catalase (E.C. 1.11.1.6) was estimated using method given by Aebi [46] with slight modifications. Catalase activity was calculated using extinction coefficient of 6.93×10^−3^ mM^−1^cm^−1^. Guaiacol peroxidase (EC 1.11.1.7) was determined by the method as given by Putter [47] with slight modifications. An extinction coefficient of 25 mM^−1^cm^−1^ was used to calculate the enzyme activity. Ascorbate peroxidase (EC 1.11.1.11) is responsible for the detoxification of peroxides such as hydrogen peroxide using ascorbate as a substrate. The specific activity was calculated using an extinction coefficient of 2.8 mM^−1^cm^−1^ [48]. Gluatathione reductase (EC 1.8.1.7) was determined by the method of Carlberg and Mannervik [49]. The enzyme activity was calculated using an extinction coefficient of 6.22 mM^−1^cm^−1^. Lactate dehydrogenase (EC 1.1.1.27, L-lactate: NAD^+^ oxidoreductase) activity was determined using oxidation rate of pyruvate at 340 nm by NADH following the method of Kuznetsov and Gnaiger [50] with slight modifications. The enzyme activity was calculated using the extinction coefficient 6.22 mM^−1^cm^−1^.

#### Effect of Erucin on DMBA Induced Alteration on Other Oxidative Stress Parameters in Liver Homogenate

Lipid peroxidation was determined using the formation of Thiobarbituric Acid Reactive Species (TBARS), lipid hydroperoxides and conjugated diene formation following the method of Devasagayam *et* al. [51] and Jiang et al. [52] respectively with slight modifications. The amount of TBARS was expressed as µmol MDA Equivalent per gram of tissue. The regression equation obtained was y = 185.9x+0.003. Lipid hydroperoxide content was expressed as mM H_2_O_2_ equivalent per gram of tissue. The amount of conjugated diene in homogenate was determined by vortexing liver homogenate with chloroform:methanol mixture (2∶1) followed by centrifugation at 2000 rpm. The lower chloroform layer obtained after discarding upper layer was dried in vacuum evaporator at 45°C. The residue was dissolved in cyclohexane and absorbance was measured at 233 nm against cyclohexane. The reduced glutathione content of the tissue was determined using the method given by Anderson [53]. The regression equation used for calculation of SH content (milli moles of SH content per gram of tissue) was y = 0.3122x+0.0035 and was obtained using reduced glutathione (GSH) as a standard.

#### Effect of Erucin on DMBA Induced Alterations in Serum Enzymes

Following treatments, 4 ml blood was taken from anaesthetized rats using retro orbital blood collection in heparinized collection tubes. These were later centrifuged at 1500 g at 4°C for 12-15 min. Serum alkaline phosphatase, serum glutamic oxaloacetic transaminase (SGOT), serum glutamic pyruvic transaminase (SGPT) and serum bilirubin were analyzed using protocols supplied by autopack kits in 96 well plates using synergy HT multi-mode microplate reader by BioTek Instruments Inc.

### Histology

Liver dissections were done and the viscera were carefully immersed in 10% formaline and transported to the histology lab. Sections were taken from various representative areas which were processed and embedded in paraffin blocks. Three to four micron thick sections from paraffin blocks were stained with haematoxylin and eosin stain. Coded histological sections of liver biopsies were scored independently by two different histopathologists using the Ishak modified histological index grading (HAI) [54]. A consensus score was calculated after discussion on the points of difference for comparison of various classification and statistical calculations.

### Statistical Analysis

The experimental data were expressed as mean ± SE. One way analysis of variance (ANOVA) and Tukey's HSD post hoc test were carried out to determine significant differences between the mean at p≤0.05.

## Supporting Information

Figure S1 Confirmation of the purity of erucin as shown by **(A)** GC-FID showing the single peak of erucin (100%), **(B)** mass spectra of erucin, **(C)** ^1^H NMR spectra at 600 MHz, **(D)** ^13^C NMR spectra at 600 MHz.
(TIF)
