# Supplementary figures and images for: Hepatic Dysfunction Induced by 7, 12-Dimethylbenz(α)anthracene and Its Obviation with Erucin Using Enzymatic and Histological Changes as Indicators
Source: PLoS One. 2014 Nov 12;9(11):e112614. doi: 10.1371/journal.pone.0112614 (PMC4229223; doi:10.1371/journal.pone.0112614)

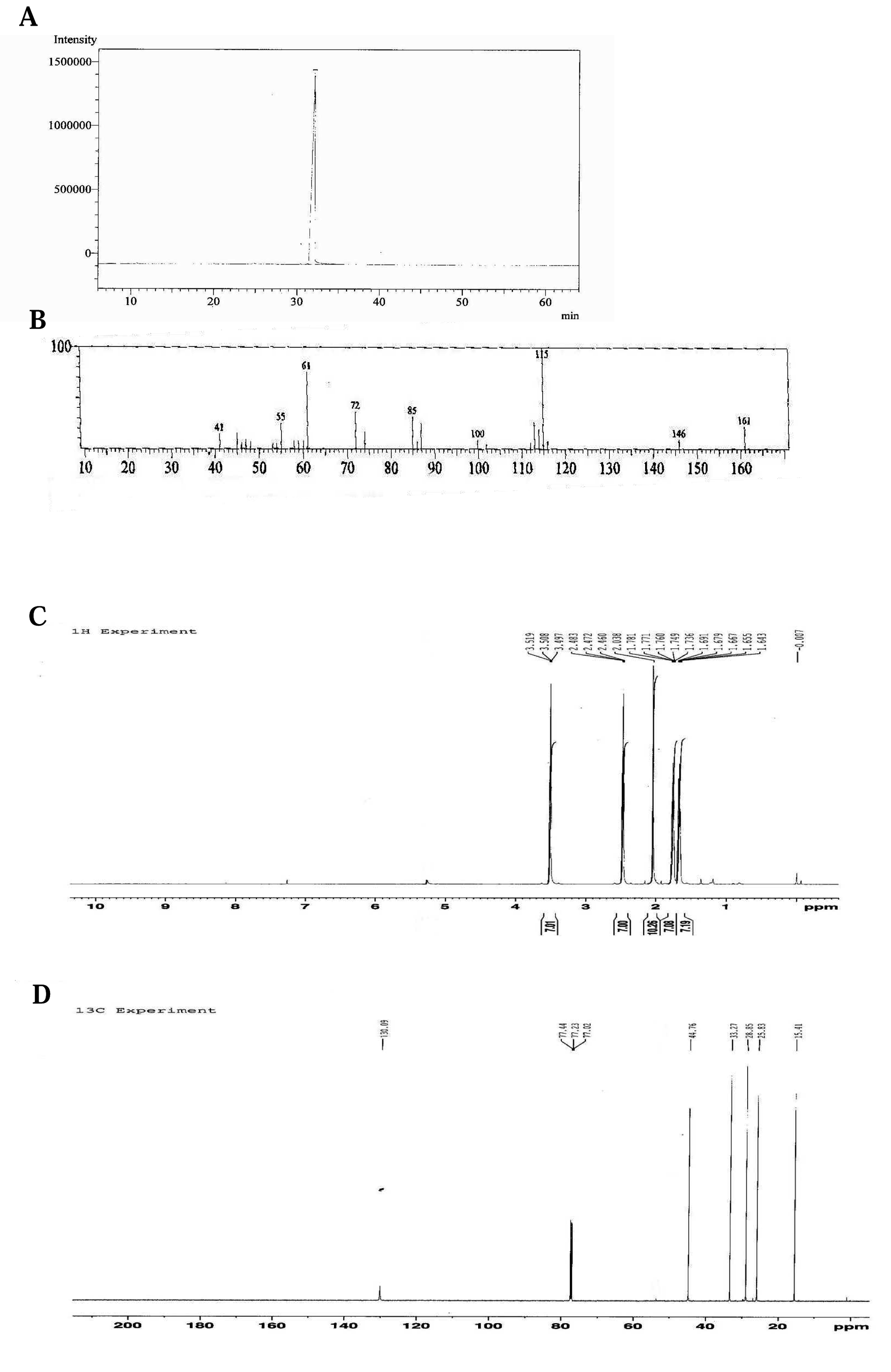

Supplement: Figure S1 — Confirmation of the purity of erucin as shown by (A) GC-FID showing the single peak of erucin (100%), (B) mass spectra of erucin, (C) 1H NMR spectra at 600 MHz, (D) 13C NMR spectra at 600 MHz. (TIF) [file pone.0112614.s001.tif]
